# Vitamin B12 deficiency: epidemiological trends and risk factors

**DOI:** 10.3389/fnut.2026.1930037

**Published:** 2026-10-16

**Authors:** Gurudayal S. Toteja, Nishant Toteja

**Affiliations:** 1 Jamia Hamdard University, New Delhi, India; 2 Department of Pediatrics, Hamdard Institute of Medical Sciences and Research, New Delhi, India

**Keywords:** cobalamin, epidemiology, India, micronutrient deficiency, one-carbon metabolism, pregnancy, vegetarian diet, vitamin B12

## Abstract

Vitamin B12 (cobalamin) can only be obtained from animal food sources and is important for several functions, including DNA synthesis, haematopoiesis and myelination. It is one of the most common micronutrient deficiencies worldwide. Accurate estimates of the global burden remain difficult to ascertain because studies differ in the biomarker used (total serum/plasma B12, holotranscobalamin, or methylmalonic acid) and in the diagnostic cut-off applied, and because countrywide, longitudinally comparable data remain limited. This narrative review conducted following a defined literature search and selection strategy described in Section 1.1—synthesizes the current epidemiology of vitamin B12 deficiency, compares India's experience with other nations, and examines how reported prevalence has changed over the past 50 years. Pooled meta-analytic estimates indicate that approximately 50% of the Indian population is affected by vitamin B12 deficiency, among the highest rates reported worldwide. Reported prevalence rises across the life course, from 14% to 17% to 31% to 48% to 62% in pre-schoolers, school-aged children, adolescents, adults and the elderly respectively, with substantial regional heterogeneity within India (Section 2.8). Prevalence among pregnant women has been reported to range from 40% to 70%. The most consistently reported associated factors are dietary pattern (vegetarianism), low socioeconomic status, advancing age and female sex; because the underlying studies are predominantly cross-sectional, these should be read as associations rather than established causal pathways. Reported prevalence has remained high across every assessment period from the 1970s to the present, though this pattern is reconstructed from independent cross-sectional studies rather than a single longitudinal cohort (Section 4). We conclude that improved, harmonized diagnostic criteria, targeted screening, and reconsideration of current food-fortification standards are needed, while noting the implementation and evidentiary challenges that fortification scale-up in India still faces (Section 5).

## 1 Introduction

Vitamin B12 is an important water-soluble vitamin synthesized only by micro-organisms (bacteria). It is found primarily in animal-derived foods, including red meat, fish, eggs, and milk, with a very small contribution from foods or water exposed to such organisms. Vitamin B12 serves as a critical co-factor in two important reactions: the conversion of homocysteine to methionine via methionine synthase, and the conversion of methylmalonyl-CoA to succinyl-CoA via methylmalonyl-CoA mutase. It is therefore essential for DNA synthesis, red cell production, and maintenance of nerve myelin. Deficiency can manifest as megaloblastic anemia and glossitis, peripheral neuropathy, subacute combined degeneration of the cord, and cognitive impairment; in pregnancy, low maternal B12 has been associated with neural tube defects and other adverse birth outcomes, as well as longer-term metabolic risk in the offspring (1, 2).

Deficiency may result from inadequate dietary intake (common in populations that avoid animal-source foods for cultural or religious reasons) or from malabsorption due to a range of conditions (atrophic gastritis, pernicious anemia, gastrointestinal infection, or prior gastrointestinal surgery); malabsorptive causes become more prevalent with age. In India, insufficient dietary intake is the most common cause.

Vitamin B12 deficiency can be assessed using several biomarkers (total serum/plasma vitamin B12, holotranscobalamin, methylmalonic acid, or homocysteine). Because total serum vitamin B12 is often not an accurate indicator of tissue-level deficiency, functional indicators such as holotranscobalamin and methylmalonic acid can provide more accurate assessment (3, 4); the choice of biomarker, together with the cut-off applied, is a major and recurring source of inconsistency between studies, a point we return to explicitly in Section 4.1.

The objective of the review is to consolidates evidence on vitamin B12 deficiency in India and compares it with other regions worldwide. It is organized in two parts: the first provides a comprehensive account of the Indian scenario by age, sex, physiological status, diet and region; the second reviews the global geographic picture and analyses reported changes in prevalence over the last 50 years.

### 1.1 Review methodology

This is a narrative rather than a systematic review, and no prospective protocol was registered, consistent with recommendations for transparent reporting of narrative reviews [the Scale for the Assessment of Narrative Review Articles, SANRA (5)]. The search of literature was made through PubMed/MEDLINE, Scopus, and Google Scholar for English-language publications from January 1966 to July 2026, using combinations of the terms “vitamin B12,” “cobalamin,” “deficiency,” “prevalence,” “epidemiology,” “India,” “pregnancy,” and “vegetarian,” together with relevant country and region names for the global comparison in Section 3. The search prioritized to systematic reviews and meta-analyses, nationally representative surveys (notably the Comprehensive National Nutrition Survey, CNNS), and large multicentre studies; where subgroup-specific national data were unavailable (e.g., for the elderly or for lactating women). These were supplemented with hospital-based and community cohort studies, identified in part by screening the reference lists of the systematic reviews retrieved (snowballing).

Studies were included if they reported the prevalence of biochemically defined vitamin B12 deficiency, by total serum/plasma B12, holotranscobalamin, or methylmalonic acid, in a human study population. Case reports, non-English-language publications, and studies that did not specify a deficiency threshold were excluded. Because this review spans multiple countries and a five-decade window, a formal PRISMA flow diagram was not applicable, selection instead relied on the authors' joint judgement of study relevance, representativeness, and methodological quality, prioritizing the largest and most representative available evidence for each subgroup and geography considered.

## 2 Epidemiological data in India

Epidemiological data from India indicate that it has one of the highest documented burdens of vitamin B12 deficiency worldwide. A systematic review and meta-analysis of 26 studies on B12 deficiency in India found an overall national prevalence of approximately 53% (95% CI, 41%−64%), among the most prevalent micronutrient deficiencies in India alongside vitamin D and iron (6). An independent meta-analysis of 20 studies (*n* = 18,750), published between 2001 and 2023, produced a pooled prevalence of approximately 51% (95% CI 44%−57%), with the burden concentrated among women, vegetarians, adolescents and pregnant women (7). The combined effects of a largely cereal-based lacto-vegetarian diet, low consumption of animal-based foods, high rates of poverty, and limited public-health prioritization are the factors most consistently proposed to explain the high prevalence of vitamin B12 deficiency in India (1, 6). The following subsections detail prevalence across population groups. The life-course pattern is summarized in Figure 1 and Table 1.

**Figure 1:**
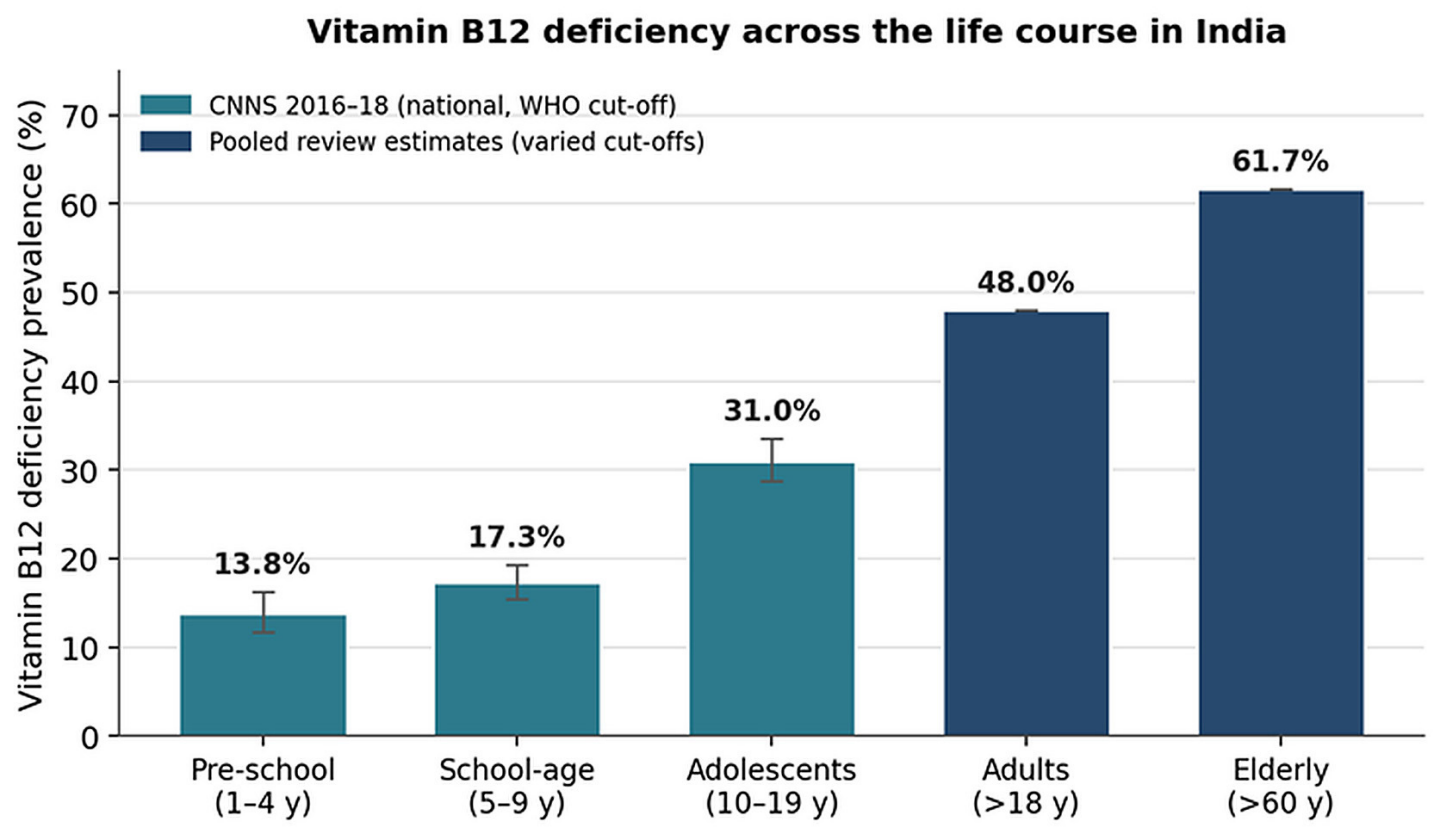
Vitamin B12 deficiency prevalence across the life course in India. Pre-school, school-age, and adolescent values are from the Comprehensive National Nutrition Survey 2016–18 (WHO cut-offs, with 95% confidence intervals); adult and elderly values are pooled estimates from review data using heterogeneous cut-offs and should be interpreted as indicative only. Sources: Venkatesh et al. (6), Shalini et al. (8), and Malik et al. (9).

**Table 1:** Summary of vitamin B12 deficiency prevalence, biomarkers/cut-offs, and key risk factors across population groups in India. Figures and cut-offs are drawn from heterogeneous source studies (see Section 4.1) and should be read as indicative rather than directly comparable point estimates.

Population group	Prevalence	Biomarker/cut-off used	Key risk factors	**Ref**.
Pre-school children (1–4 y)	13.8% (95% CI 11.7–16.2)	Serum B12, WHO cut-off	None significant by sex	(8)
School-age children (5–9 y)	17.3% (95% CI 15.4–19.3)	Serum B12, WHO cut-off	Rural residence (local studies)	(8, 10)
Adolescents (10–19 y)	31.0% national (28.7–33.5); up to 42.7% in central region; 57% in pooled 0–17 y estimate	Serum B12, WHO cut-off/varied cut-offs	Male sex, rural residence	(6, 8, 10)
Adults (>18 y), general population	48% pooled (35%−62%); 24.2% in a large urban health-check cohort	Serum B12; cut-offs from < 150 pmol/L to < 211 pg/mL	Vegetarian diet, low socioeconomic status, female sex, advancing age	(6, 9, 11, 13)
Elderly (>60 y)	61.7% pooled (varied cut-offs); 22% (M)/9.5% (F) in one urban cohort	Total serum B12, varied cut-offs	Atrophic gastritis, H. pylori infection, poor nutrition	(4, 9)
Pregnant women	40%−70% range; ~67% pooled estimate	Serum B12	Vegetarian diet, low socioeconomic status	(1, 7)
Lactating women/breastfed infants	Elevated risk (not separately quantified nationally)	Maternal serum B12; breast-milk B12	Low maternal B12 stores, no B12 in antenatal supplementation	(1)
Healthy young vegetarian adults	~50% deficient by serum; 70% (M)/50% (F) with low holotranscobalamin; 92% (M)/50% (F) with hyperhomocysteinaemia	Serum B12, holotranscobalamin, homocysteine	Strict lacto-vegetarian diet	(3)

### 2.1 Children (male and female)

The Comprehensive National Nutrition Survey (CNNS, 2016–2018), covering children and adolescents from every state, produced the first nationally representative estimates of pediatric B12 status. Using WHO criteria, 13.8% (95% CI 11.7–16.2) of pre-school (1–4 y) children and 17.3% (95% CI 15.4–19.3) of school-age (5–9 y) children were B12 deficient, with little difference between boys and girls at these ages (8). Smaller cohort and hospital-based studies report wide variation in prevalence, generally attributed to local dietary patterns and infectious disease burden.

### 2.2 Adolescent girls and boys

Adolescence, alongside early childhood, is a period of particular vulnerability. In the CNNS, 31.0% (95% CI 28.7–33.5) of adolescents aged 10–19 years were B12 deficient (8), roughly double the school-age rate. A pooled meta-analytic estimate for the 0–17-year age band was higher, at 57%, though based on fewer studies and with a wider confidence interval (6). In a cross-sectional study of healthy school-going adolescents, approximately 32% were deficient, with boys more often affected than girls, and rural adolescents more often affected than urban adolescents (10).

### 2.3 Adult men and women

The pooled prevalence of vitamin B12 deficiency in adults (>18 years) across studies in the largest meta-analysis was 48% (95% CI 35–62%) (6); one Indian study reported deficiency as high as 78.5% in healthy adults, depending on the serum cut-off used (9). Landmark studies from Pune measuring cobalamin, homocysteine and methylmalonic acid in Asian Indian men and women documented a markedly high prevalence of biochemical B12 deficiency (11). Clinical data from northern India have repeatedly shown an adult prevalence around 47% (12). In a large multicentre study of over 15,000 asymptomatic adults undergoing routine health checks across seven northern Indian states, the overall prevalence using a serum cut-off of 211 pg/mL was 24.2%, with advancing age and female sex identified as significant risk factors (13); as discussed in Section 4.1, this considerably lower figure reflects both a higher cut-off and a more affluent, self-selected sample rather than a true reduction in burden. Comparatively, healthy Indian women have shown higher serum B12 concentrations, and lower deficiency prevalence, than healthy Indian men in some cohorts (9).

### 2.4 Elderly men and women

Older adults may be at elevated risk of B12 deficiency because of poor nutrition and age-associated reductions in absorption (e.g., atrophic gastritis, reduced gastric acid secretion, Helicobacter pylori infection). A review of Indian adults and elderly individuals reported 61.7% deficiency in the elderly using varied cut-offs (9). A study of apparently healthy urban elderly Indians measuring total B12 found 22% deficiency in men and 9.5% in women (4). Hospital series show the number of diagnosed cases increasing with age, peaking around 50–65 years (14, 15). Internationally, elderly prevalence is reported at 10%−15%, and as high as 31% in some countries (8); these figures remain considerably lower than those observed in India, reflecting differences in diet and in the underlying causes of deficiency.

### 2.5 Pregnant and lactating women

A synthesis of 46 studies on B12 status among pregnant and lactating women in India found deficiency prevalence ranging from 40% to 70% (1), and a meta-analysis estimated approximately 67% of pregnant women to be affected (7). International meta-analyses have similarly found insufficient B12 during pregnancy to be common globally and associated with lower birth weight (2). In Indian cohorts, low maternal B12 alongside adequate-to-high folate has been associated with neural tube defects, recurrent pregnancy loss, gestational diabetes, pre-eclampsia, intrauterine growth restriction, and adverse metabolic and cognitive outcomes in the child; because these data derive from observational cohorts, they should be interpreted as associations rather than as demonstrated causal pathways (1). The risk of deficiency persists into lactation, since low maternal stores lower breast-milk B12 and place exclusively breastfed infants at risk; current Indian antenatal policy supplements only iron and folate, not vitamin B12 (1).

### 2.6 Diet (lacto-vegetarian and mixed)

Diet is the single most important determinant of B12 status in India. Individuals consuming a lacto-vegetarian diet, a substantial share of the Indian population, have significantly lower cobalamin levels than those consuming a mixed diet (6, 9, 11). In one study of healthy young vegetarian adults, approximately 50% had serum B12 below the deficiency threshold, and 70% of men and 50% of women had low holotranscobalamin; in contrast, approximately 92% of men and 50% of women had hyperhomocysteinaemia (3), illustrating that different biomarkers can yield materially different deficiency estimates within the same cohort (see Section 4.1). Reviews consistently show several-fold higher deficiency among lacto-vegetarians than non-vegetarians (6, 9), and dietary insufficiency, not pernicious anemia, is the dominant cause of B12 deficiency in India (1). Dietary fortification and diversification through dairy and meat consumption have been shown to improve B12 status (6, 9).

### 2.7 Rural and urban populations

Studies from Pune comparing middle-aged men in rural, slum, and middle-class urban settings found a high frequency of low B12 (and elevated homocysteine) across all three settings, with rural and slum residents faring worse than urban middle-class residents (16). Among adolescents, rural residents also showed higher deficiency prevalence than urban residents (10). Conversely, research from a small “tier-3” town in North India found higher deficiency prevalence than among urban endocrine-clinic patients, suggesting that socioeconomic status and health-seeking behavior can alter the expected urban–rural gradient (12). Pooled analyses indicate that rural-vs.-urban differences in B12 status are inconsistent across studies (7); overall, dietary intake and socioeconomic status appear to be stronger determinants of B12 status than geographic setting *per se*.

### 2.8 Regional heterogeneity within India

In the CNNS, B12 deficiency prevalence was highest in the central region of India across every pediatric age band assessed-−21.0% (95% CI 14.9–28.7) in pre-schoolers, 29.1% (95% CI 24.5–34.2) in school-age children, and 42.7% (95% CI 37.4–48.2) in adolescents—each substantially above the corresponding national estimates reported in Sections 2.1–2.2 (8). Despite this marked regional gradient, the same survey found no consistent difference in B12 deficiency between rural and urban locations at the national level (8), a pattern that sits alongside, and partly explains, the inconsistent and sometimes contradictory rural/urban findings from individual state-level studies discussed in Section 2.7. Adult-level state-by-state prevalence data comparable in scale to the CNNS are not currently available, which we note as a specific gap for future national surveillance (Section 5).

## 3 Global epidemiological data (geographic)

Outside India, B12 deficiency follows clear geographic and economic lines, high-income populations with adequate access to animal-source foods have lower deficiency prevalence, while low- and middle-income populations are more affected, owing to both scarcity of animal-source foods and a higher burden of malabsorptive conditions. A widely cited compilation of national and regional datasets found serum B12 deficiency (below 130–150 pmol/L) in 11%−15% of women in Germany and the United Kingdom; 31% of elderly individuals in the UK; 49% of adults in India; 40% of children in Kenya; 28% of pregnant women in Nepal; and 20% of the same demographic group in New Zealand (8). Figure 2 compares these geographic estimates.

**Figure 2:**
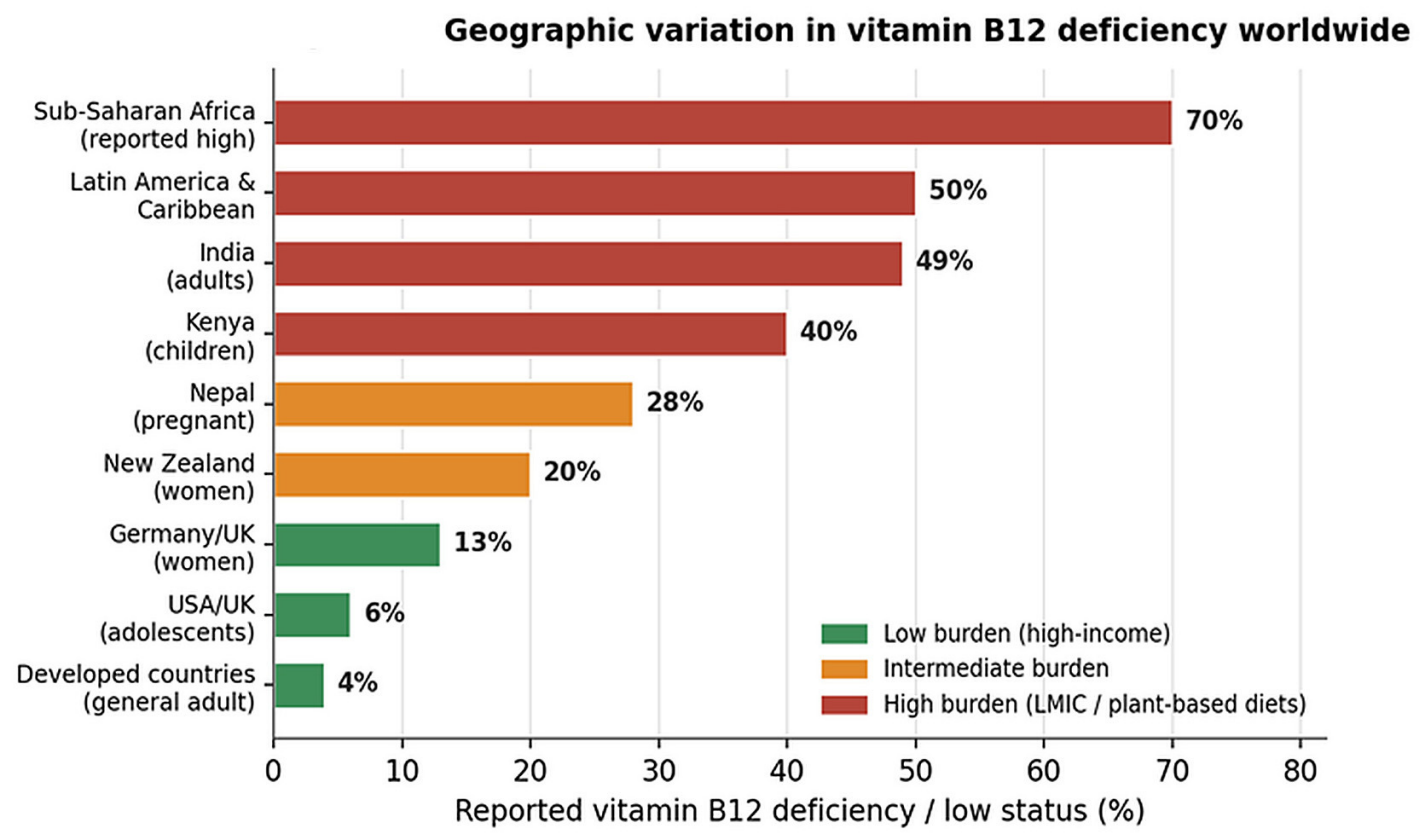
Geographic variation in reported vitamin B12 deficiency/low status. Figures are drawn from comparative survey reviews and represent different population groups (general adults, adolescents, women, pregnant women or children) and diagnostic thresholds; they are therefore indicative rather than strictly comparable. Sources: Venkatesh et al. (6), Shalini et al. (8), Gebremedhin et al. (17), and regional surveys cited therein.

### 3.1 North America and Europe (high income)

Clinical B12 deficiency in adults in North America and Europe is uncommon, generally reported at 3%−5% (and up to 10%−15% in older individuals); 31% of older UK adults have been reported as deficient (8). Around 6% of adolescents are affected in both the United States and the United Kingdom. Malabsorptive causes (pernicious anemia, atrophic gastritis, and drug-induced malabsorption from metformin or proton-pump inhibitors) remain important, though the growing popularity of strict vegan and vegetarian diets is adding a dietary component to deficiency in these regions (6). Pernicious anemia remains a recognized cause of megaloblastic anemia in populations of European and African ancestry (6).

### 3.2 Latin America and the Caribbean

Systematic reviews of national and public-health surveys in this region show a high prevalence of low or marginal B12 stores, with more than 40% of individuals affected by clinical or subclinical deficiency in many settings; an analysis of 41 studies suggested up to 61% of individuals in Mexico and Central/South America had inadequate B12 stores (17). Limited access to animal-source foods, combined with high rates of gastrointestinal infection, is cited as a key driver (6).

### 3.3 Sub-Saharan Africa

Africa has the fewest epidemiological studies and consequently the least high-quality prevalence data. Available national micronutrient surveys indicate 54%, 35%, and 15% B12 deficiency among reproductive-age women in Malawi, Kenya, and Ethiopia respectively, with a lower prevalence (7%) in Ghana; some school-based studies report figures approaching 70% (8, 17). Insufficient animal-source food consumption, food insecurity, and gastrointestinal/parasitic infection burden are the principal contributing factors (17).

### 3.4 Asia-Pacific and eastern Mediterranean

The Indian subcontinent and neighboring South Asian countries report the highest deficiency prevalence worldwide, 70%−80% in some series, primarily reflecting a predominantly vegetarian, carbohydrate-based diet (6). Nepal, for example, reports approximately 28% deficiency among pregnant women (8); Bhutanese and South Asian migrant populations also show high prevalence. Rates are lower among East and South-East Asian populations with greater fish and animal-food consumption, except among vegetarians within those populations (6). In high-income Asia-Pacific settings such as New Zealand, approximately 20% of women are deficient (8). The Eastern Mediterranean region shows highly variable prevalence, with deficiency recognized as a regional problem wherever animal-product consumption is low.

## 4 Trend analysis of the last 50 years and its interpretation

A key constraint on reconstructing a 50-year trend in vitamin B12 deficiency is that no single longitudinal, nationally representative cobalamin survey exists in India, or indeed anywhere else, spanning this period. Most available estimates are cross-sectional snapshots using different biomarkers, cut-offs, and populations. The analysis below is therefore a compilation of representative estimates by time period (Figure 3), reflecting measured prevalence and the growing research attention to this issue over time, rather than a single internally consistent cohort followed longitudinally.

**Figure 3:**
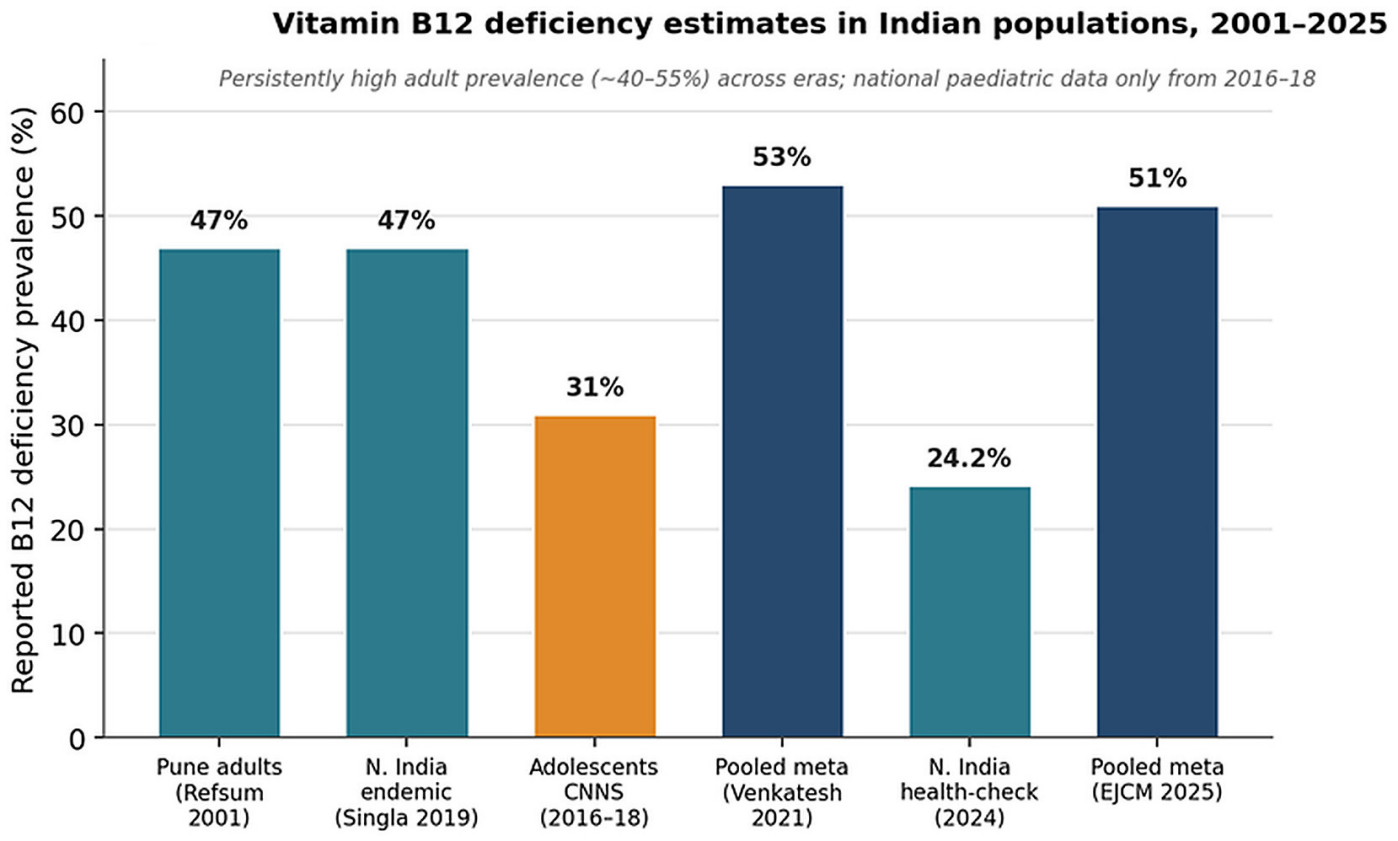
Reported vitamin B12 deficiency prevalence in landmark Indian studies and meta-analyses, 2001–2025, within a five-decade research horizon. Each bar is a published estimate; the populations and cut-offs differ (teal = adult/general clinic or biomarker study; orange = national adolescent survey; navy = pooled meta-analysis), so bars are not strictly comparable. The 2024 health-check value is lower because of a higher serum threshold and an affluent, self-selected urban sample. Sources: Venkatesh et al. (6), Shalini et al. (8), Singla et al. (12), Refsum et al. (11), Gupta et al. (13), and Dubey et al. (7).

Evidence from the 1970s and 1980s was largely clinical and descriptive: early recognition that asymptomatic Indian lacto-vegetarians had lower B12 than non-vegetarian counterparts, and observations of anemia among Asian immigrants in London, raised the possibility of a population-level problem, but few surveys used contemporary assays to quantify it. Systematic biochemical assessment began at the turn of the millennium with the Pune studies (Refsum, Yajnik et al., 2001–2006), which documented a high prevalence of low cobalamin with concomitantly elevated homocysteine across sampled Indian populations (11, 16).

Since 2000, the evidence base has grown substantially. Clinic and community studies through the 2010s consistently showed an adult prevalence of approximately 47% (12); the CNNS (2016–2018) provided the first national pediatric and adolescent estimates (14%−31%) (8); and two independent meta-analyses synthesizing studies to 2020 and 2023 reported pooled adult/general prevalence of 51%−53% (6, 7). A large 2024 health-check dataset reported a considerably lower figure of 24.2%, which, as discussed in Section 4.1, most plausibly reflects a higher serum cut-off and a more affluent, urban, self-selected sample rather than a genuine decline (13).

### 4.1 Comparability limitations across biomarkers and cut-offs

A limitation running through every prevalence estimate cited in this review is the absence of biomarker and cut-off harmonization. Total serum/plasma B12 thresholds used across the cited studies range from below 150 pmol/L to below 211 pg/mL (13), and most studies rely on total B12 rather than on functional markers such as holotranscobalamin or methylmalonic acid, which more directly reflect tissue cobalamin status (3, 4). Total serum B12 is itself an indirect marker: it can be influenced by recent dietary intake, by pregnancy-related haemodilution, and by inter-laboratory assay variability. A concrete illustration of the resulting inconsistency comes from a single cohort of healthy young Indian vegetarians, in which roughly 50% were deficient by total serum B12, but 70% of men and 50% of women had low holotranscobalamin, and 92% of men and 50% of women had hyperhomocysteinaemia (3). Therefore a 20-percentage-point-or-greater spread in “deficiency” depending solely on which biomarker was used. Consequently, the pooled and range estimates presented throughout this review, and summarized in Figures 1–3 and Table 1, should be read as indicative bands reflecting the underlying heterogeneity of methods rather than as directly interchangeable point estimates; differences between studies (for example, the 24.2% vs. 51–53% adult estimates discussed above) reflect this methodological variability at least as much as any true difference in underlying prevalence.

### 4.2 Publication bias and evidence quality

The largest pooled meta-analysis underlying the national prevalence estimates cited in this review formally assessed small-study (publication bias) effects across the six micronutrient deficiencies it examined, including vitamin B12, using funnel plots and Egger's test, and found no statistically significant asymmetry (*p* > 0.05) (6). This could be reassuring rather than conclusive. Egger's test has limited power when the number of contributing studies is modest, as is the case for several of the population subgroups discussed here (e.g., the elderly and lactating women), and selective emphasis on statistically significant subgroup findings within published studies cannot be fully excluded. Most of the underlying primary studies are single-center, hospital- or community-based cross-sectional surveys; while several used the NIH or Joanna Briggs Institute quality-assessment tools for observational studies, formal risk-of-bias grading was not uniformly reported. The diagnostic heterogeneity (Section 4.1) has been flagged and this residual uncertainty around bias assessment as the two principal limitations that should temper strong quantitative interpretation of any single prevalence figure in this review.

### 4.3 Interpretation

Three patterns emerge from the data. First, persistence with adult/general-population prevalence in India has remained high, in the region of 40%−55%, across every period for which data exist, with no discernible decline over the past 25 years despite rising national wealth and dietary change (6, 7, 11, 12); this conclusion rests on convergence across independently conducted cross-sectional studies rather than repeated measurement of a single cohort, and should be read with that caveat. Second, expanding epidemiological recognition: enhanced measurement efforts, exemplified by the CNNS, have progressively closed long-standing data gaps, particularly for children and adolescents (8). Third, methodological variability: differences between surveys (for example, 24.2% from the 2024 health-check sample vs. 51%−53% from pooled meta-analyses) are driven substantially by differences in cut-points, biomarkers, and sampling frames (Section 4.1), in addition to any true temporal change (6, 13). Globally, a parallel trend is the growing recognition of B12 deficiency as a major population-level dietary problem, not only among older adults with pernicious anemia in wealthy countries, but increasingly in lower- and middle-income countries adopting more plant-based diets (6, 17). Overall, the epidemiological picture is one of a large, stable, and under-addressed chronic burden rather than a rapidly changing epidemic; continued improvement in standardized, harmonized dietary and biochemical surveillance will be needed to determine whether this pattern is genuinely stable or whether it is chiefly an artifact of persistently heterogeneous measurement.

## 5 Summary and conclusion

Table 1 summarizes reported prevalence, biomarkers/cut-offs, and principal risk factors across the population groups discussed in this review.

Vitamin B12 deficiency is one of today's most frequent micronutrient deficiencies worldwide. In India, the deficiency rate is markedly higher (51%−53%) (6, 7) than the 3%−6% rates reported among high-income adults and adolescents (8). Reported prevalence increases across the life course, from around 14% in early childhood to 31% in adolescence, 48%−62% among adults and older adults, and 40%−70% during pregnancy (1, 6, 8, 9), with substantial regional heterogeneity within India that headline national figures can obscure (Section 2.8). Modifiable dietary patterns, principally the lacto-vegetarian, cereal-based diet with limited animal-food intake, together with low socioeconomic status, female sex, older age, and age-related malabsorption, are the most consistently reported associated factors (3, 6, 9, 13), given the predominantly cross-sectional, observational nature of the underlying evidence, these are best characterized as associations rather than confirmed causal mechanisms. Geographic patterns broadly parallel access to animal-source foods and the burden of malabsorptive and infectious disease, lower prevalence in North America and Europe, higher prevalence in South Asia, Latin America, and sub-Saharan Africa (6, 8, 17).

Two limitations apply across the estimates discussed in this review. The first is diagnostic heterogeneity, the absence of consensus on biomarkers and cut-off points introduces imprecision and contributes to the wide variation observed across studies (Section 4.1). The second is the absence of a reliable, repeated, longitudinal survey from which long-term trends can be determined with confidence, for India, the apparent 50-year time series is a derived composite of many independent cross-sectional studies rather than a true cohort (Section 4). In addition, the related limitation is that formal assessment of small-study/publication bias has so far been reported for only one of the pooled meta-analyses cited, and did not reach statistical significance, but cannot be assumed to generalize to every subgroup discussed (Section 4.2). Despite these caveats, the weight of the evidence is consistent and supports the review's central conclusion regarding the scale of the problem.

From a public health perspective, it is important to establish harmonized diagnostic criteria for B12 deficiency, ideally combining total circulating B12 with a functional marker such as holotranscobalamin or methylmalonic acid. Routine screening is recommended particularly for pregnant and lactating women, adolescents, the elderly, and strict vegans/vegetarians (3, 4). Evidence-based strategies to reduce this long-standing burden include dietary diversification, food fortification, and the addition of B12 to existing antenatal and adolescent supplementation programmes, which currently contain only iron and folic acid (1, 6). On fortification specifically, the evidence is directionally supportive but implementation-dependent. India's current (2018) FSSAI fortification standards for folic acid and vitamin B12 are set at roughly 90% below WHO-recommended levels for Indian consumption patterns, having been lowered from stronger 2016 standards (18), and a recent review identifies concrete barriers to more effective scale-up, including uneven regulatory enforcement, continued reliance on voluntary fortification of open-market atta, variable miller-level capacity and quality assurance, and insufficient long-term monitoring of nutritional outcomes among beneficiaries (19). A that large-scale staple fortification has also drawn published concerns from Indian public-health researchers regarding the strength of the underlying evidence base and potential safety considerations at higher fortification levels (20); any revision of national standards should therefore be accompanied by the monitoring and evaluation infrastructure needed to track both benefit and safety at population scale. Regional data gaps, particularly the absence of CNNS-scale state-level surveillance in adults (Section 2.8), should also be prioritized in future national nutrition surveys.

To summarize, vitamin B12 deficiency is a largely preventable, straightforward-to-treat disorder, and its continued neglect in India's public health programming is difficult to attribute to anything other than insufficient prioritization of the condition relative to its documented scale.
